# Circulating tumor DNA: from discovery to clinical application in breast cancer

**DOI:** 10.3389/fimmu.2024.1355887

**Published:** 2024-04-30

**Authors:** Jiachi Xu, Hongyu Gao, Xinyu Guan, Jiahao Meng, Shirong Ding, Qian Long, Wenjun Yi

**Affiliations:** ^1^ Department of General Surgery, The Second Xiangya Hospital, Central South University, Changsha, Hunan, China; ^2^ Clinical Research Center For Breast Disease In Hunan Province, Changsha, China; ^3^ Department of Oncology, The Second Xiangya Hospital of Central South University, Changsha, Hunan, China

**Keywords:** breast cancer, circulating tumor DNA, screening, diagnosis, prognosis, treatment

## Abstract

Breast cancer (BC) stands out as the cancer with the highest incidence of morbidity and mortality among women worldwide, and its incidence rate is currently trending upwards. Improving the efficiency of breast cancer diagnosis and treatment is crucial, as it can effectively reduce the disease burden. Circulating tumor DNA (ctDNA) originates from the release of tumor cells and plays a pivotal role in the occurrence, development, and metastasis of breast cancer. In recent years, the widespread application of high-throughput analytical technology has made ctDNA a promising biomarker for early cancer detection, monitoring minimal residual disease, early recurrence monitoring, and predicting treatment outcomes. ctDNA-based approaches can effectively compensate for the shortcomings of traditional screening and monitoring methods, which fail to provide real-time information and prospective guidance for breast cancer diagnosis and treatment. This review summarizes the applications of ctDNA in various aspects of breast cancer, including screening, diagnosis, prognosis, treatment, and follow-up. It highlights the current research status in this field and emphasizes the potential for future large-scale clinical applications of ctDNA-based approaches.

## 1 Introduction

Breast cancer (BC) stands out as the cancer with the highest incidence of morbidity and mortality among women worldwide, and its incidence rate is currently trending upwards (1). While advancements in surgical treatments, chemotherapy, radiotherapy, immunotherapy, and other therapeutic modalities have exerted some control over breast cancer mortality (2), a substantial number of patients who have undergone well-regulated systematic treatments still succumb to tumor progression (3). Hence, there is a pressing need to enhance breast cancer screening methods and bolster the efficacy of diagnosis and treatment. Given the complex nature of drug development, we find ourselves compelled to shift our focus toward revisiting traditional perspectives on screening and treatment. It is imperative that we explore new avenues to improve breast cancer screening and diagnostic precision, as well as the effectiveness of treatment, in order to reduce the toll this disease exacts on patients.

Traditional screening methods have predominantly relied on imaging techniques. However, there is a prevailing perception that organized screening of young, healthy women may be inefficient and potentially harmful (4), despite the fact that breast cancer mortality rates are notably higher in this demographic (5). It’s worth noting that the decline in breast cancer mortality owes itself more to changes in risk factors and improved treatments than to advancements in imaging-based screening approaches (6). Furthermore, the current diagnosis and treatment of breast cancer heavily rely on invasive tissue biopsies and immunohistochemistry (7). These procedures are commonly performed by the operator under local anesthesia, using a coarse needle puncture to obtain a small piece of the patient’s tumor tissue, or through surgical excision of the mass. Immunohistochemical staining of well-established markers is then conducted to classify the type of breast cancer and develop an appropriate treatment plan. Imaging studies of other parts of the body and individual characteristics are also taken into consideration during this process (8). Nevertheless, breast cancer exhibits significant heterogeneity as a malignant tumor, evolving in various directions under the influence of diverse drivers. This is especially pronounced in advanced breast cancers that have metastasized to distant sites, where different tumor foci may exhibit entirely distinct molecular features (9–11). Consequently, relying solely on small tissue samples obtained through punctures or separate tissue sections is grossly inadequate in capturing the full extent of this heterogeneity (12). While some guidelines advocate for biopsying distant metastases, practical difficulties often arise in implementation (13, 14). Additionally, there is the inherent risk of misjudging the assessment of distant metastases through imaging alone. Given these challenges, many treatment options may warrant reconsideration from the outset. Furthermore, in terms of prognostic evaluation, despite numerous clinical studies offering survival statistics based on patients’ baseline characteristics, the inherent tumor heterogeneity and intrinsic variations among patients can lead to starkly different survival outcomes, even among those with early-stage breast cancer (15). This underscores the pressing need for more precise prognostic indicators that can better predict patients’ survival outcomes.

In addition to these aforementioned shortcomings, current methods for monitoring breast cancer treatment have their own set of limitations. Presently, the monitoring and follow-up of breast cancer treatment heavily rely on a series of computed tomography (CT) scans (16), which are not without their issues. Firstly, there can be a delay in the regularity of testing. If a drug-resistant clone is present in a patient, it may have significantly progressed by the time it becomes discernible through imaging (17). Secondly, the phenomenon of pseudoprogression can further complicate matters by leading to unnecessary additional treatments (18). Most concerning of all, repeated exposure to radiation through these scans not only elevates the radiation burden on the patient but also poses challenges in assessing bone metastases (19).

To overcome these shortcomings, a portion of brand-new markers have been partially validated. These new methods are usually defined as multiparametric, multianalytical and multigene assays. Some of these markers have been recommended by experts for use in clinical practice. Oncotype DX, MammaPrint and uPA/PAI-1 are some of the validated assays (20–22). Although the clinical efficacy of these markers has been demonstrated, they are expensive and, moreover, these methods require tumor tissue. Due to the limitations of tumor tissue, some circulating biomarkers have been explored, such as tissue polypeptide-specific antigen(TPS), carcinoembryonic antigen(CEA) and carbohydrate antigen 15.3(CA 15.3), which unfortunately are not sensitive enough to be widely used in clinical practice (23–25). It is therefore particularly important to find markers that are more minimally invasive and reflect the full extent of the tumor landscape.

Circulating tumor DNA (ctDNA) emerges as a compelling solution to address these critical questions. As a part of the library of cell free DNA (cfDNA) released after apoptosis or necrosis throughout the body (26), it can provide a more comprehensive genetic map of tumors and heterogeneity landscape at the molecular level (19). Remarkably, this can be achieved with a nearly non-invasive approach, yielding substantial benefits throughout the entire spectrum of breast cancer diagnosis and treatment. In our comprehensive review, we begin with an overview of the discovery of ctDNA and the way in which its detection content is used in breast cancer. Subsequently, we delve into an extensive examination of ctDNA’s pivotal role in the entire breast cancer diagnosis and treatment continuum, spanning screening, diagnosis, prognosis, treatment, and post-treatment follow-up. We aim to shed light on the potential clinical applicability and scalability of ctDNA, envisioning its widespread adoption in clinical practice. We compared the advantages and disadvantages of ctDNA with traditional diagnostic methods in breast cancer examination and treatment, as shown in **Table 1**.

**Table 1:** Comparison of ctDNA with traditional methods.

Method	Advantages	Disadvantages
ctDNA	Minimally invasive, no radioactive contamination, new DNA no preservative contamination Early diagnosis or prediction of recurrence Real-time monitoring of treatment response Determining survival expectations and risk of recurrence in tumor patients	Cannot be detected in some patients with early or advanced cancers Limited sensitivity and specificity Undiagnosed
Imaging	Tumor location and size can be localized Minimally invasive	Difficulty in detecting microscopic lesions Possible false positives and negatives
Tissue biopsy	Tumor typing can be determined	Invasive testing
Tumor marker	Low cost of testing, minimally invasive	Poor sensitivity and specificity

## 2 Discovery and content of circular DNA

The existence of extracellular nucleic acids in the bloodstream was initially uncovered as far back as 1948 by Mandel et al (27). However, it wasn’t until three decades later, in 1977, that LEON et al. made a pivotal breakthrough. They showed that concentrations of circulating DNA were notably elevated in patients with metastatic cancer compared to healthy individuals. This discovery was groundbreaking as it highlighted that tumor cells, in their course of undergoing apoptosis or necrosis, release the DNA fragments they contain into the circulatory system. This release leads to a rise in circulating DNA levels, which is closely linked to the effectiveness of anticancer treatments (28).

**Figure 1:**
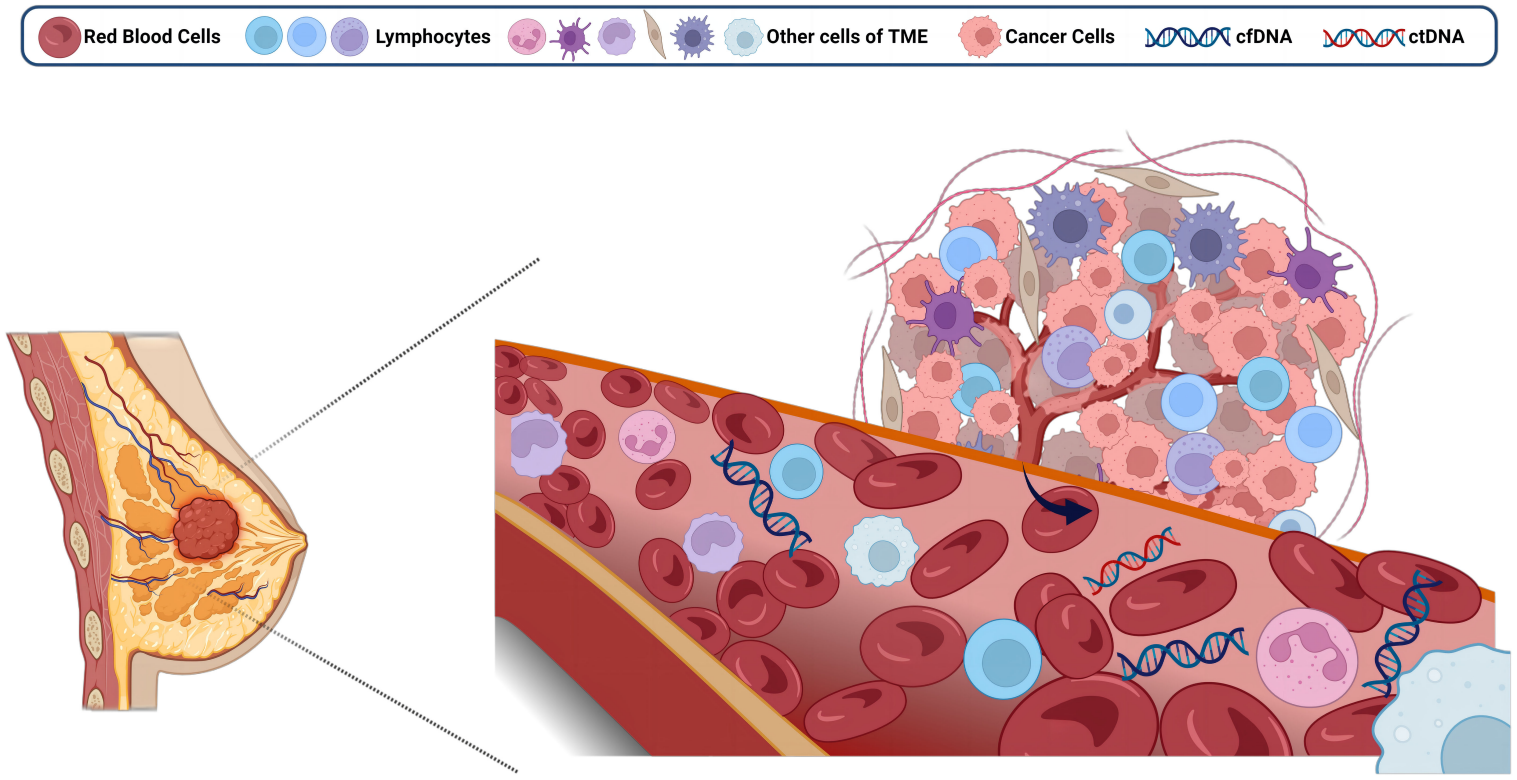
Free DNA from the breast tumor microenvironment enters the circulation. Created with BioRender.com.

In healthy individuals, cfDNA (70-90%) is mainly derived from neutrophils and lymphocytes (29). However, in cancer patients, the principal contributors to cfDNA are the components of the tumor microenvironment (TME), which include tumor cells and tumor-infiltrating T lymphocytes (30) (**Figure 1**). Of these, only cfDNA derived specifically from tumor cells is referred to as ctDNA (31). Distinguishing ctDNA from cfDNA was historically quite challenging, as ctDNA could account for as little as 0.01% of the total cfDNA pool (32). Consequently, in the early stages of research, the detection of ctDNA was relatively rare, with the primary focus centered on cfDNA. Accurate quantification of ctDNA under various conditions has been achieved through diverse methods, such as the deconvolution algorithm (33), solid-state nanopores (34). However, widespread application remains hindered by cost and technological constraints.

The results of multiple dimensions of the ctDNA test content can be a key tool in the diagnosis and treatment of breast cancer. As DNA fragments produced by cancer cells during necrosis and apoptosis (26), the number of mutant ctDNA molecules in plasma is mainly proportional to the tumor burden (7). Moreover, the value/concentration of cfDNA/ctDNA can be precisely measured, making it a major target for quantitative studies. As detection technology continues to advance, including methods like DNA methylation (35), microsatellite instability (MSI) (36–38), loss of heterozygosity (LOH) (39), DNA integrity (DI) (40), and others based on epigenetic and global alterations in cancer DNA, there is an expanding toolkit for detecting and quantifying these modifications. These innovative techniques can play a crucial role in the diagnosis and treatment of breast cancer, contributing as quantitative parameters in research studies.

**Figure 2:**
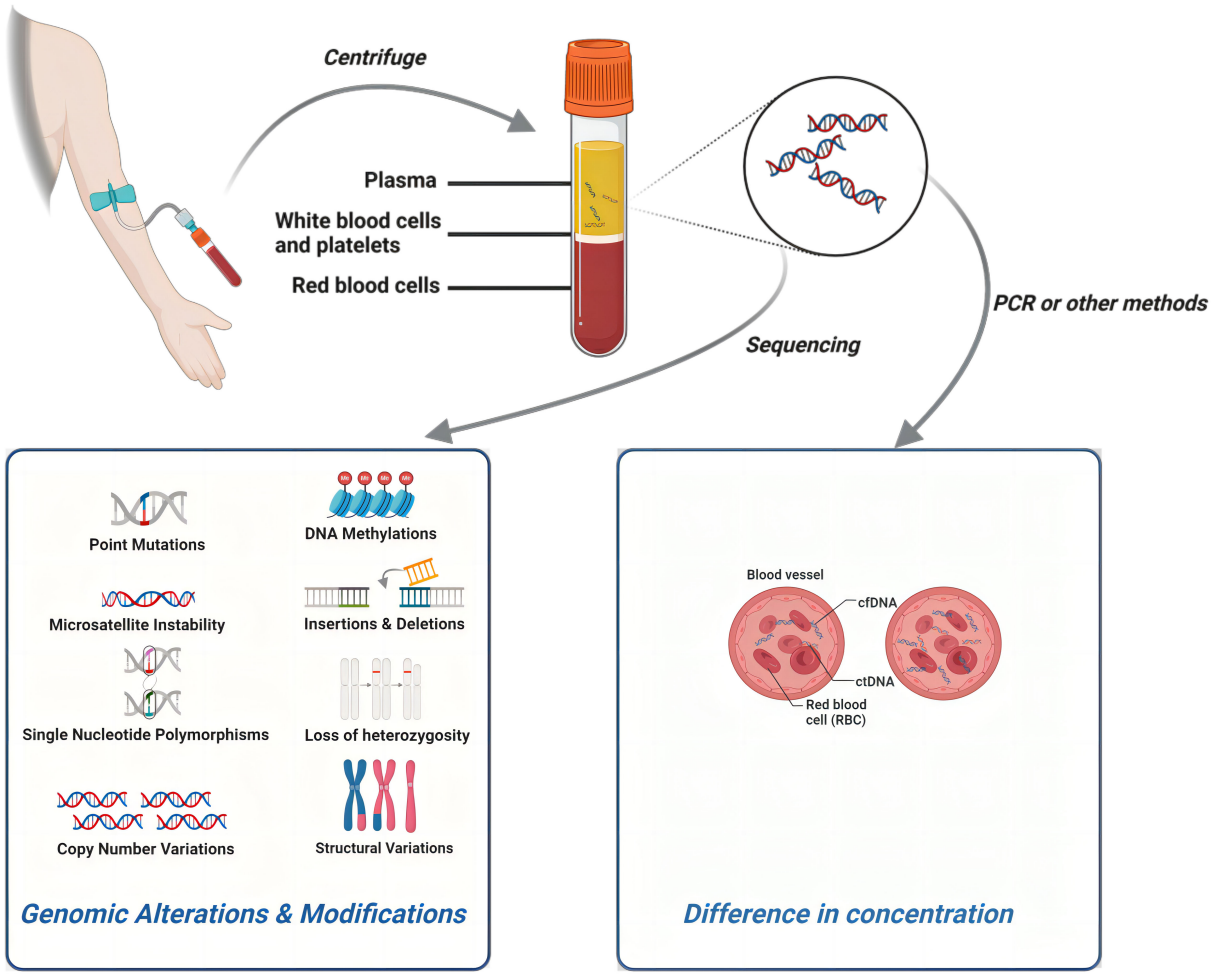
The detection process of ctDNA and different research directions within ctDNA can all play a role in breast cancer. Created with BioRender.com.

Tumor-specific mutations, as elucidated through comprehensive ctDNA sequencing, wield significant influence over the diagnosis and treatment of breast cancer. The specific genes, genomic loci, and mutation types, encompassing single nucleotide polymorphisms (SNPs) and copy number variations (CNVs), among others, along with the diverse spectrum of DNA epigenetic modifications mentioned earlier, exert a multifaceted impact on the behavior of breast cancer. Consequently, they emerge as focal points in various qualitative studies. Certain classic genes and pathways, such as TP53, PIK3CA, and ESR1 (41–43), have gained widespread recognition as pivotal prognostic and therapeutic markers. It is anticipated that these markers will see expanded adoption in clinical settings in the foreseeable future, paving the way for broader clinical utilization (**Figure 2**).

Another avenue for leveraging ctDNA in the diagnosis and treatment of breast cancer is through the development of models based on the factors mentioned above. These models often exhibit enhanced efficiency and predictive quality. Some of the particularly potent models are poised for further scrutiny and validation in larger-scale clinical studies, promising to enhance our understanding of their clinical utility.

## 3 ctDNA and breast cancer screening

Disease screening demands comprehensive coverage of the population while maintaining a delicate balance between accuracy, convenience, affordability, and minimal invasiveness. Hence, non-compliant tests are typically unsuitable for screening unless their efficacy significantly surpasses that of established conventional tests. The prevailing standard for breast cancer screening is mammography (44), known to reduce mortality rates by up to 30% (45). However, mammography is not infallible, with a sensitivity that falls short of 100%, resulting in approximately 13% of breast cancer cases being missed (46). Moreover, false positives can lead to unwarranted invasive procedures and the potential for radiation-induced health issues (47). Additionally, the compression of tumors during mammographic procedures can elevate central venous blood ctDNA levels. Although this may not necessarily lead to adverse clinical outcomes, it can introduce variability in the standardization of circulating tumor marker sampling procedures, rendering results less reliable (48). Furthermore, it’s crucial to acknowledge that it takes a significant period for cancer cells to evolve and coalesce into detectable solid tumors, which can contribute to delays in diagnosis and subsequent treatment initiation.

cfDNA/ctDNA emerges as a potent complement to address the aforementioned issues. In earlier investigations, Huang et al. conducted a comparison of plasma cfDNA concentrations among breast cancer patients, individuals with benign breast tumors, and a normal control cohort. They unearthed a striking finding: the median cfDNA concentration in breast cancer patients was approximately fivefold higher than that in the healthy control group (with medians of 65 ng/mL and 13 ng/mL, respectively). Of note, within this study, the median cfDNA concentration in the malignant tumor group was thrice as high as that in the benign breast tumor group (with medians of 65 ng/mL and 22 ng/mL, respectively). This suggests that there was a significant difference of plasma DNA values among healthy individuals, NBC patients, and BCa patients (p=0.001) (49). This result underscores the presence of a discernible difference in cfDNA concentration between the two groups, even among individuals generally regarded as healthy. This seems to suggest that the tumor tissue itself, whether benign or malignant, will have a higher metabolism and consequently release more cfDNA into the circulation, whereas malignant tumors may have a much higher rate of cfDNA release. This may be due to the fact that malignant tumors have more active cell proliferation activity, leading to more tumor cell death and DNA release. In contrast, benign tumors usually grow at a slower rate and have less cell proliferation activity, so the concentration of cfDNA may be relatively low. Indeed, similar results have been demonstrated in studies of other malignant tumors.

A study by Xie et al. on ctDNA in non-small cell lung cancer and benign lung tumors also showed that cfDNA concentrations were significantly higher in lung cancer patients than in the benign group, and that there was a positive correlation between cfDNA levels and tumor size or maximal tumor diameter (r=0.430, P=0.022) (50). In a similar study, median cfDNA concentrations were significantly higher in patients with primary colorectal cancer than in normal controls and patients with intestinal polyps) (51). This pattern of elevated cfDNA levels has also been corroborated across different stages of breast cancer. Leon et al. conducted a study revealing that plasma cfDNA levels were markedly higher in patients with advanced breast cancer compared to those with early-stage breast cancer (28). Tangvarasittichai et al. conducted a quantitative study, further reinforcing this correlation by establishing that plasma DNA concentration positively correlates with the stage of breast cancer (52). Moreover, advancements in cfDNA detection methods hold the potential to redefine the future of breast cancer screening. A meta-analysis study demonstrated that six studies utilizing contemporary qualitative cfDNA detection methods achieved superior mean sensitivity and specificity (0.88 and 0.98, respectively) compared to digital mammography (with sensitivity and specificity values of 0.87 and 0.89, respectively). Notably, digital mammography is currently the most widely employed method for breast cancer screening (53).

Beyond direct utilization of cfDNA concentration, certain DNA modifications, like DNA methylation, offer valuable complementary tools for early breast cancer screening. Investigations targeting DNA methylation typically employ two primary strategies: (1) untargeted screening, which assesses global DNA methylation, and (2) assays designed to examine methylation patterns in specific genes. Moreover, these studies often differentiate between samples obtained from whole blood and plasma. In a previous systematic review, it was observed that BRCA1 and RASSF1A were the most frequently investigated genes in whole blood and plasma, respectively. Importantly, the frequency of methylation in these genes was notably higher among breast cancer patients (54). Nevertheless, it’s worth noting that some studies have pointed to a positive correlation between whole-gene hypomethylation and an increased risk of breast cancer (55, 56). This discrepancy may arise from differences in the populations studied, emphasizing the need for more extensive and rigorously designed research to draw conclusive distinctions. More recently, findings from Xu et al. have hinted that DNA methylation profiles in blood begin to exhibit changes years before clinical detection of breast cancer (57). However, further evidence is required before it can be officially recognized as a breast cancer screening marker. Nevertheless, risk stratification several years in advance offers valuable insights into which populations may benefit from more frequent screening protocols.

Qualitative analysis of cfDNA in healthy individuals offers valuable insights into risk stratification for breast cancer. In a prolonged follow-up investigation involving liquid biopsies among healthy individuals, a noteworthy observation emerged: four initially healthy blood donors with detectable oncogenic mutations eventually developed benign tumors or invasive breast cancers within the subsequent 1 to 10 years of follow-up (58). This intriguing finding suggests that if larger prospective studies were to scrutinize the effectiveness of liquid biopsy as a clinical tool for breast cancer screening, it might pave the way for more proactive cancer management and improved prognoses within this seemingly “healthy” population.

ctDNA also holds promise for breast cancer screening during pregnancy, offering an alternative for expectant mothers who may be hesitant to undergo imaging-related tests due to concerns about fetal health. Lenaerts et al. conducted an investigation into the sensitivity of the genome-wide routine noninvasive prenatal testing (NIPT) pipeline in detecting cancer cell-specific copy number alterations (CNAs) in ctDNA from breast cancer patients. To mitigate the influence of fetal cfDNA, they organized different study groups. The results revealed that the sensitivity to detect specific CNAs was 36% in the pregnant group, significantly higher than the 16% sensitivity observed in the non-pregnant group. Remarkably, 15% of the tested cases were asymptomatic at the time of blood collection (59). While it’s important to note that the sensitivity achieved is not exceptionally high, the marked difference between the two groups provides evidence supporting the use of ctDNA for breast cancer screening during pregnancy.

## 4 ctDNA and breast cancer diagnosis

The current diagnosis of breast cancer heavily relies on pathology, and our focus here centers on the pivotal role of cf/ctDNA in the diagnostic landscape of breast cancer. Pathological diagnosis necessitates invasive procedures, guided by medical imaging equipment, and it stands as the established “gold standard” for diagnosing breast cancer (60). Consequently, when evaluating the worth of cf/ctDNA in breast cancer diagnosis, the first consideration is its unique capacity to address challenges that pathology currently cannot overcome. Although pathology appears to be all-encompassing, there exists a subset of cases known as occult breast cancer—instances where no lesion is detectable through imaging. In these perplexing scenarios, biopsy-based pathologic diagnosis proves inadequate. Typically, patients with occult breast cancer present with an axillary mass as their initial complaint, often accompanied by lymph node metastases that signify an locally advanced stage of the disease (61). In patients with occult malignant tumors whose primary site is unknown, cf/ctDNA can identify the presence of cancer (62), which will greatly improve the prognosis of such patients.

To expand the application of ctDNA in breast cancer (BC) diagnosis on a larger scale, it’s essential to scrutinize its diagnostic capabilities. A meta-analysis conducted by Lin et al. offers valuable insights, revealing that, in the 24 studies included, the mean sensitivity and specificity of cfDNA as a diagnostic tool were 0.7 and 0.87, respectively. However, in a more recent and extensive meta-analysis encompassing 29 studies on breast cancer diagnosis, the sensitivity and specificity demonstrated remarkable improvement, reaching 80% and 88%, respectively (53). This notable enhancement could be attributed to advancements in assay technology and underscores the considerable potential of cfDNA/ctDNA as a standalone marker for breast cancer diagnosis.

The outcomes of qualitative studies are amenable to quantification, with a predominant focus on DNA modifications and aberrant gene expression. In the previously mentioned study by LIN et al., which incorporated 10 qualitative analyses (including methylation PCR, microsatellite analysis, and sequencing), the sensitivities and specificities for breast cancer diagnosis were 0.50 and 0.98, respectively (53). This trend persisted in the follow-up study, underlining the significance of qualitative cfDNA testing, particularly in evaluating gene-specific methylation status as a supplementary tool to enhance the specificity of breast cancer diagnosis (63). Notably, the parameter cfDI, formulated based on cfDNA integrity, exhibited superior diagnostic efficacy for early-stage breast cancer compared to other liquid biopsy results, including CTCs, cfDNA concentration, or CA153 (64). Another study showed that the incidence of LOH was significantly correlated with lymph node status in terms of the relative concentration of DNA in preoperative serum in patients with breast cancer, in patients with benign breast disease, and in healthy women (36). In addition, a study of the differences in MSI and LOH between breast cancer and healthy women showed significant differences in some alleles (65). These collective findings underscore the value of comprehensive ctDNA studies in the early diagnosis of breast cancer.

The diagnosis of advanced breast cancer assumes equal importance. A noteworthy ctDNA investigation pertaining to breast cancer leptomeningeal metastasis (BCLM) showcased compelling results. Specifically, quantifying cerebrospinal fluid (CSF) ctDNA in the study’s participants achieved a remarkable 100% sensitivity and specificity in diagnosing BCLM, surpassing the traditional “gold standard” CSF cytology (66). Furthermore, the inclusion of measurements for cfDNA aneuploidy mutations in CSF contributed to enhanced diagnostic efficiency (67). In another study, it was observed that the ctDNA fraction exhibited a notable elevation up to 12 weeks prior to the clinical progression of BCLM (68). These findings highlight the critical role of ctDNA in the diagnosis of advanced breast cancer, especially in cases where meningeal metastases occur.

## 5 ctDNA and breast cancer prognosis

In conventional perception, the prognosis of breast cancer primarily revolves around its classification and tumor staging. BC patients with similar typing and staging have highly variable prognoses, which may be mainly related to tumor heterogeneity. Tumor heterogeneity prevents a single tumor sample obtained by biopsy from being fully used to identify genomic alterations (10, 69). This highlights the need for additional biomarkers to more accurately stratify recurrence risk (70). On the other hand, the identification of minimal residual disease (MRD) is important because MRD is highly associated with a high risk of recurrence of BC (71–73). ctDNA is theorized to be a collection of exfoliated DNA from the entirety of tumor cells, and thus in can compensate for the shortcomings of traditional tissue biopsies; and because of its molecular properties, it can also be used as a tool for early detection of MRD. In the subsequent sections, we delve into a comprehensive exploration of studies involving ctDNA as a prognostic marker for breast cancer, highlighting its diverse applications in various contexts.

In a study involving patients with early-stage breast cancer who had undergone immediate surgical intervention, Olsson et al. made a significant observation. They found that ctDNA testing demonstrated exceptional accuracy in identifying post-surgical recurrence, boasting a sensitivity of 93% and a specificity of 100%. Furthermore, the median lead time for clinical detection of recurrence was 7.9 months (71). These findings underscore the potential of ctDNA as a highly effective tool for early detection of recurrent breast cancer in patients who have undergone surgical treatment.

Results in BC patients undergoing neoadjuvant therapy (NAT) have been notably diverse and revealing. In a study conducted by Garcia-Murillas et al., involving 55 breast cancer patients receiving neoadjuvant chemotherapy (NAC), plasma ctDNA testing yielded remarkable insights. It accurately predicted metastatic recurrence, both at specific time points and during continuous follow-up, showcasing its potential as a valuable prognostic tool (74). Furthermore, a comprehensive meta-analysis examining the correlation between baseline ctDNA and survival outcomes in early-stage breast cancer unveiled compelling findings. Notably, the presence of ctDNA at baseline, preceding NAT, was linked to a significant decline in both relapse-free survival (RFS) and overall survival (OS) (75). These findings underscore the valuable role of ctDNA in assessing treatment response and prognosis in breast cancer patients undergoing neoadjuvant therapy.

Enhancing the ability to predict treatment response during neoadjuvant therapy represents a valuable complement to current treatment approaches. The conventional measure of pathologic clinical remission (pCR) based on imaging assessment is often regarded as the gold standard for prognosis in breast cancer patients undergoing neoadjuvant therapy (76). However, it is prone to inaccuracies. In this context, ctDNA emerges as a novel and promising marker for monitoring neoadjuvant therapy. Multiple studies have highlighted that continuous monitoring of ctDNA during neoadjuvant therapy offers valuable insights. Specifically, when ctDNA is cleared during the course of neoadjuvant therapy, it is associated with improved RFS in these patients (77, 78). Conversely, individuals in whom ctDNA remains detectable after the completion of neoadjuvant therapy or surgery are at a higher risk of recurrence and tend to exhibit poorer OS outcomes (73, 74, 79). This underscores the potential of ctDNA as a dynamic and responsive marker for tracking treatment response during neoadjuvant therapy in breast cancer cases.

Additional targeted studies have delved into the use of ctDNA as a prognostic marker for BC across different molecular subtypes. In one such investigation, which focused on the relationship between ctDNA and prognostic indicators in triple-negative breast cancer (TNBC) patients who had undergone neoadjuvant chemotherapy as part of the BRE12-158 clinical trial (ClinicalTrials.gov Identifier: NCT02101385), compelling findings emerged. It was observed that the detection of ctDNA significantly correlated with poorer distant disease-free survival (DDFS), disease-free survival (DFS), and OS outcomes (80). A similar study of patients with hormone receptor (HR)-positive/HER2-negative breast cancer and TNBC receiving neoadjuvant chemotherapy in the I-SPY2 (ClinicalTrials.gov Identifier: NCT01042379) trial noted that early clearance of ctDNA 3 weeks after the start of therapy predicted the degree of sensitivity to NAC in patients with TNBC and that ctDNA positivity was associated with reduced distant recurrence-free survival in both subtypes sensitivity to NAC in TNBC patients, and that ctDNA positivity was associated with reduced distant recurrence-free survival in both subtypes. As this was a longitudinal surveillance study, results after NAC treatment showed that ctDNA negativity was associated with improved prognosis (81). These consistent findings across different molecular subtypes underscore the broad applicability and potential of ctDNA as a robust prognostic marker for breast cancer patients.

ctDNA also demonstrates a robust association with prognosis in advanced breast cancer. Insights from a real-world study focused on advanced breast cancer revealed a substantial correlation between a circulating tumor fraction (TF) of ≥10%, calculated using single-nucleotide polymorphism aneuploidy across the ctDNA genome, and OS (82). Notably, specific mutated genes play pivotal roles in this context. Among the genomic alterations present in cfDNA and tumor DNA (tDNA) in high-risk stage 3 and 4 breast cancer patients, TP53 and PIK3CA mutations, along with epidermal growth factor receptor (EGFR) and ERBB2 amplifications, emerged as the most common. TP53 mutations (p = 0.0004) and PIK3CA mutation allele frequency [p = 0.01, HR 1.074 (95% CI 1.018-1.134)] were particularly strong predictors of progression-free survival (PFS) (83). In another extensive retrospective study involving patients with locally advanced breast cancer and metastatic breast cancer (MBC), the mean percentage of ctDNA was found to be 4.5% (ranging from 0 to 88.2%), with the number of variants averaging 3 (ranging from 0 to 27). TP53 (52%), PIK3CA (40%), and ERBB2 (20%) were the most frequently affected genes. Significantly, differences in PFS and OS were statistically significant when comparing patients with baseline ctDNA percentages <0.5% and ≥0.5% (P=0.003 and P=0.012, respectively) (84). These findings underscore the substantial prognostic value of ctDNA in advanced breast cancer cases.

Several biomarker-specific studies have brought attention to this phenomenon. One noteworthy example is the TBCRC 005 study, a prospective investigation focused on biomarkers. This study employed an innovative quantitative multiplex assay known as cMethDNA to identify a novel set of cfDNA methylation markers in the plasma of metastatic breast cancer (MBC) patients. It then generated a cumulative methylation index (CMI) based on six out of the ten genes detected. Remarkably, high CMI levels in patients exhibited a significant correlation with both shorter median survival and OS (85). Recently, the study published an update in which a novel liquid biopsy-breast cancer methylation combination marker was collected from 144 MBC patients at baseline, week 4, and week 8, and the median PFS and OS were significantly shorter in MBC patients with high cumulative methylation (CM) compared to those with low cumulative methylation. Based on the circulating CM levels at week 4, an effective model was developed that allows for the prediction of disease progression after three months, as early as week 4 after the start of a new treatment in MBC patients (86). This will greatly advance the timing of patients changing treatment regimens and provides evidence for the large-scale application of ctDNA as a prognostic marker.

## 6 ctDNA and breast cancer treatment

Typically, the treatment plans for BC patients are carefully crafted by considering the gene expression patterns, molecular characteristics of the tumor specimen, as well as Supplementary Information such as imaging results from other sites and individual patient attributes (8). These attributes encompass factors like TNM staging, tumor grade, and receptor expression status. Breast cancer itself is classified into four intrinsic subtypes: luminal A, luminal B, HER2-enriched, and TNBC. These subtypes exhibit markedly distinct treatment regimens, molecular features, and biological characteristics (87, 88).

Some patients do not have satisfactory treatment results, which may be due to (1) the fact that some molecular information is not available because of the heterogeneity within the tumor, and (2) the emergence of new resistant clones with new driver mutations due to tumor evolution, leading to the fact that new targeted therapies against a particular locus may only be efficacious for a short period of time (89). Indeed, cancers accumulate somatic mutations as they evolve (90). Some of these mutations act as drivers that lead to clonal expansion (91, 92). Mutations, in combination with factors such as spatial segregation, lead to the formation of genetically distinct cell populations that express different oncological characteristics, termed intra-tumor heterogeneity (ITH) (93). The existence of ITH has been widely accepted (94, 95). Studies have shown that sequencing of both breast cancer and metastases across time and space reflects great heterogeneity (96). In fact, ITH is a key factor contributing to mortality, treatment failure and drug resistance in breast cancer (97). This has led to confusion about the current way of diagnosing breast cancer: is focal, single histologic examination the optimal solution to reflect molecular information about breast cancer?

Furthermore, *de novo* mutations that arise during the clinical progression of tumors frequently result in resistance to targeted therapies (98). A study conducted by Kim et al., which focused on the driver genes associated with chemoresistance in TNBC, demonstrated that resistance genes are already present at an early stage and undergo adaptive selection throughout the course of chemotherapy (99). This is in contrast to *de novo* mutations emerging midway through chemotherapy. Consequently, there is a pressing need for the rapid and reliable identification of these novel driver mutations to enable more timely and precise treatment strategies.

### 6.1 Tracking heterogeneity among breast cancers and tumor clonal evolution using ctDNA

There is unquestionably significant value in employing ctDNA testing as a complement to traditional pathology-based diagnostic methods. ctDNA has the capacity to provide a comprehensive molecular profile of breast cancer, playing a pivotal role in unraveling the mechanisms governing tumorigenesis, progression, metastasis, and the development of drug resistance. ctDNA serves various crucial functions in the detection and analysis of breast tumors, spanning their development and progression (100). Firstly, ctDNA has the remarkable capability to capture the heterogeneity within breast cancer and monitor the real-time evolution of tumor clones. This was vividly illustrated by Murtaza et al., who demonstrated that all mutations present in primary tumors and metastatic lesions could be identified in ctDNA (101). In addition, They followed multiple tumor and plasma DNA samples from a patient with MBC for 3 years and found that all metastatic-grade mutations that initially appeared in biopsy samples from the primary tumor were detected in plasma samples, and that backbone and metastatic mutations were identified by looking at the category of samples in which they appeared (metastatic foci or all samples) (102). This insight provides invaluable insights into the evolutionary trajectory of cancer clones. This means that the detection of ctDNA is able to reflect a comprehensive genetic map of cancer, including the clonal hierarchy identified by multiregional tumor sequencing, and allows the tracking of different treatment responses in different metastases. This is important for the timely detection, quantification and tracking of cancer progression and recurrence processes in order to adopt the most accurate and timely means of responding to events during cancer treatment and follow-up. In addition, the identification of mutational priorities could update the understanding of targets and perhaps allow the design of new models to reclassify subgroups of patients to achieve “accurate and on-time” therapeutic outcomes.

### 6.2 Therapeutic guidance for ctDNA in breast cancer

Exome sequencing does have its limitations, particularly in patients with a low tumor burden and reduced plasma ctDNA levels, primarily due to its lower coverage compared to targeted depth sequencing. In contrast, targeted depth sequencing exhibits superior performance in identifying low-frequency variants within the specified target region, making it better suited for analyzing low-quality samples in clinical settings (103). However, it’s important to acknowledge that targeted sequencing, by its very nature, focuses on a selected subset of genes with established clinical relevance, potentially omitting crucial information. Therefore, careful consideration is essential when deciding on the appropriate sequencing approach. Certain genes and mutations exert a significant influence on breast cancer behavior, and we present findings from specific clinical studies to underscore the role of ctDNA testing in guiding breast cancer treatment.

Estrogen receptor (ER)-positive, HER2-negative breast cancer subtypes are the most prevalent among patients with advanced breast cancer (104). ESR1, the gene encoding the estrogen receptor, is expressed in roughly 70% of breast cancer cases (105). Resistance to aromatase inhibitors (AI) is often associated with activating mutations in ESR1 (the gene encoding estrogen receptor α, the major isoform of the estrogen receptor) in cancer subclones (106). Through cfDNA analysis, it has been revealed that ESR1 mutations can be identified in the blood of approximately 40% of patients with ER-positive, HER2-negative advanced breast cancer subsequent to the administration of aromatase inhibitors (referred to as bESR1mut) (107). Therefore, the question arises: can we predict in advance whether patients with detectable bESR1mut will develop resistance to AIs and intervene proactively?

The PADA-1 study (ClinicalTrials.gov identifier: NCT03079011) represents a randomized, open-label, multicenter phase III trial involving patients receiving AI and palbociclib as first-line therapy for metastatic ER+/HER2- breast cancer. In this study, patients will undergo periodic testing for circulating ESR1 gene mutations. Those who detect elevated circulating ESR1 mutations but do not experience tumor progression will be randomly assigned equally to one of two groups: (1) Group A, where there is no change in the original therapy, and (2) Group B, where palbociclib is combined with the selective ER downregulator fulvestrant. Recent results from this study suggest that early targeted therapy for bESR1 mutations can yield significant clinical benefits (43, 106). Another study involving estrogen receptor-positive advanced breast cancer resistant to AI treatment explored the effects of such treatment. This study, known as SoFEA (Study of Faslodex versus Exemestane with or without Arimidex, ClinicalTrials.gov identifier: NCT00253422), and PALOMA3 (Palbociclib Combined with Fulvestrant in HR-Positive/HER2-Negative Metastatic Breast Cancer after Endocrine Failure, ClinicalTrials.gov identifier: NCT01942135), involved two phase III randomized trials that assessed the impact of plasma ESR1 mutations on the sensitivity of standard therapies. These trials demonstrated that therapeutic interventions targeting patients with ESR1 mutations effectively improved PFS (108). These studies have provided evidence for the involvement of specific mutations in ctDNA in the treatment of breast cancer and have led to looking at more genes that have not been studied on a large scale, with a view to obtaining entirely new therapeutic targets.

It’s time for whole-exome sequencing(WES) to make its mark. O’Leary et al. performed WES and targeted sequencing of day 1 and end-of-treatment paired ctDNAs obtained from patients in the PALOMA-3 study and found that, in addition to the ESR1 mutation, several mutations may be associated with the development of endocrine drug resistance, and these mutations may also be involved in mechanisms related to resistance to palbociclib and fulvestrant (109). Understanding these genes is important to intervene in therapeutic regimens, but more rigorous clinical trials must be designed, and perhaps some of these genes will be the star targets of the future. We list some of the clinical trials with results in **Table 2**.

**Table 2:** Clinical trial studies with results related to ctDNA in breast cancer patients.

ClinicalTrials.gov Identifier	Clinical trial title	Participants	Results First Posted	Last Update Posted	Acronym/Other IDs
NCT00253422	Fulvestrant With or Without Anastrozole or Exemestane Alone in Treating Postmenopausal Women With Locally Advanced or Metastatic Breast Cancer	750		2011/5/17	SoFEA
NCT02181101	Simultaneous Study of Gemcitabine-Docetaxel Combination Adjuvant Treatment, as Well as Extended Bisphosphonate and Surveillance-Trial	3754		2014/7/3	SUCCESS
NCT01923168	Study of Letrozole With or Without BYL719 or Buparlisib, for the Neoadjuvant Treatment of Postmenopausal Women	340	2018/7/4	2018/9/14	NEO-ORB
NCT01633060	A Phase III Study of BKM120 With Fulvestrant in Patients With HR+,HER2-, AI Treated, Locally Advanced or Metastatic Breast Cancer Who Progressed on or After mTORi	432	2018/9/20	2019/1/30	BELLE-3
NCT02379247	BYL719 and Nab-Paclitaxel in Locally Recurrent or Metastatic HER-2 Negative Breast Cancer	43	2021/5/7	2022/6/7	CBYL719XUS06T
NCT01942135	Palbociclib (PD-0332991) Combined With Fulvestrant In Hormone Receptor+ HER2-Negative Metastatic Breast Cancer After Endocrine Failure (PALOMA-3)	521	2015/12/3	2023/4/27	PALOMA3
NCT01042379	I-SPY TRIAL: Neoadjuvant and Personalized Adaptive Novel Agents to Treat Breast Cancer	5000		2023/7/27	I-SPY2
NCT02101385	Randomized Controlled Trial of Genomically Directed Therapy in Patients With Triple Negative Breast Cancer	193	2022/10/17	2023/9/28	BRE12-158
NCT04576455	A Study Evaluating the Efficacy and Safety of Giredestrant Compared With Physician’s Choice of Endocrine Monotherapy in Participants With Previously Treated Estrogen Receptor-Positive, HER2-Negative Locally Advanced or Metastatic Breast Cancer (acelERA Breast Cancer)	303	2023/2/13	2023/11/7	acelERA BC
NCT02437318	Study Assessing the Efficacy and Safety of Alpelisib Plus Fulvestrant in Men and Postmenopausal Women With Advanced Breast Cancer Which Progressed on or After Aromatase Inhibitor Treatment.	572	2019/6/17	2023/11/18	SOLAR-1
NCT03079011	PAlbociclib and Circulating Tumor DNA for ESR1 Mutation Detection	1017	2017/3/14		PADA-1

In addition to the effects of mutations on phenotype in classical genetics, epigenetic alterations is equally crucial in life activities. DNA methylation is the most intensively studied type of epigenetics, and in general, if methylation occurs at the promoter, it may silence the gene and thus render it non-functional (110). Therefore, methylation analysis of key genes against ctDNA may have critical guidance for the treatment of breast cancer. Harvey-Jones et al. performed ctDNA longitudinal mutation and methylation analyses on homologous recombination-deficient treatment-resistant breast cancer patients and found that BRCA1 promoter methylation was detected in all plasma samples available for testing, suggesting a possible silencing mechanism for the non-rearranged BRCA1 allele in the tumors of this patient. Moreover, significant differences in BRCA1 promoter methylation were found not only in different surveillance nodes, but also in ctDNA and corresponding solid tumors (111). This suggests that BRCA1 expression is restored by an unknown mechanism, explaining the mechanism by which drug resistance arises in some breast cancers. Another study found that ESR1 epigenetic status, as assessed by methylation-specific ddPCR, can be used as an indicator of resistance to endocrine therapy in breast cancer (112). These studies highlight the role of the epigenetic status of ctDNA in guiding breast cancer treatment and have important implications for clinical management and trial design in breast cancer.

### 6.3 ctDNA can be used as a marker for immunotherapy in breast cancer

Immunotherapy has a clear role in the treatment of both early and advanced breast cancer, but it is not suitable for all patients. This is due to the high cost of immunotherapy and the equally wide variation in efficacy between individuals. ctDNA’s role in immunotherapy for breast cancer is equally diverse, and the evidence covers a wide range of types and stages. Several studies have examined ctDNA as a prognostic marker in breast cancer treated with immunotherapy. In the aforementioned I-SPY2 trial, ctDNA detection rates declined over time in both the pembrolizumab-added-to-standard neoadjuvant chemotherapy group and in the control group, and the probability of obtaining preoperative ctDNA clearance in all patients with a pCR was 100 percent (113). CtDNA can also predict prognosis in advanced breast cancer INSPIRE (ClinicalTrials. gov Identifier: NCT02644369) is a prospective phase 2 clinical study designed to evaluate the performance of a customized amplicon based ctDNA assay in predicting response in patients with advanced solid tumors treated with pembrolizumab. After analyzing ctDNA levels at baseline and at the start of cycle 3 of pembrolizumab treatment, the researchers found that in 106 patients with advanced solid tumors, including 18 patients with TNBC, lower ctDNA levels after treatment were directly associated with better OS and PFS (114).

## 7 ctDNA and breast cancer follow-up

The follow-up strategies for breast cancer patients at different stages vary. In the case of early-stage breast cancer, the primary goal is the early detection of locoregional recurrence in the affected or contralateral breast, and it typically doesn’t involve the detection of asymptomatic distant metastases. Traditional diagnostic methods, such as imaging (e.g., chest X-rays, abdominal ultrasounds, bone scans) and the use of circulating tumor markers (like carcinoembryonic antigen 15.3 and carcinoembryonic antigen), have not shown significant benefits in terms of overall survival or quality of life in this context (115). Therefore, there’s a need for the development of new tools to enhance surveillance and detect early recurrences in asymptomatic early-stage breast cancer. In a real-world study focused on the time to postoperative recurrence in early-stage TNBC patients who underwent neoadjuvant therapy and surgery, Rocca et al. made an interesting discovery. They found that in nearly all evaluated cases (with the exception of a few cases involving bone or liver metastases), ctDNA became detectable before any signs of disease spread. Moreover, the average time from ctDNA detection to the identification of suspicious findings on follow-up imaging was approximately 3.81 months, with a mean time to a definitive recurrence diagnosis of 8 months (116). Similarly, another study involving serial ctDNA testing in patients with early-stage breast cancer who had undergone neoadjuvant chemotherapy (post/preoperative) showed that the detection of ctDNA during follow-up was strongly associated with disease recurrence. Moreover, 22 of 23 patients (96%) with distant extracranial metastatic relapse could be detected by ctDNA testing (72). These studies emphasize that ctDNA is sufficiently sensitive to be of value in early-stage breast cancer follow-up.

Studies targeting ctDNA content can similarly predict recurrence. In a study of ctDNA methylation patterns in 419 cases of breast cancer in the SUCCESS study (simultaneous study of gemcitabine-Docetaxel combination adjuvant treatment, ClinicalTrials.gov identifier: NCT02181101), the serum DNAme region called EFC#93 was found to have 88% specificity in the diagnosis of metastatic breast cancer (117). It provides a new tool for predicting metastatic breast cancer in advance.

In advanced breast cancer, ctDNA can play an equally important function. The genetic map of breast cancer that has evolved through clonal selection and evolution is quite different, and continuous ctDNA concentrations and details can predict which patients should continue to be followed up, and even identify specific treatment options (118). A longitudinal ctDNA monitoring study of ER+/HER2- MBC patients was performed in which ESR1, PIK3CA, ERBB2, PTEN, TP53, KRAS, HRAS, NRAS, and AR were sequenced and analyzed. In this study, the number of ctDNA mutations was significantly associated with worsening of PFS and OS. In addition, mutation status against single genes ESR1 and TP53 predicted PFS (p = 0.024 and p = 0.035, respectively) and OS (p < 0.001 and p = 0.035, respectively) (119). These results emphasize the value of ctDNA mutation analysis in the follow-up of advanced breast cancer.

Assessing tumor heterogeneity is crucial for follow-up as higher heterogeneity in breast cancer is linked to poorer survival outcomes (120). A method called PyClone, a Bayesian clustering approach enabling the grouping of deep-sequenced somatic mutations into putative clonal clusters, can quantify tumor heterogeneity reflected by ctDNA (121). Samples with more clusters are associated with higher heterogeneity. Ma et al. collected consecutive plasma ctDNAs from 37 HER2-positive patients with metastatic breast cancer over the course of disease progression and analyzed their clonal population structure with PyClone and defined that the cluster with the highest cellular prevalence at baseline was referred to as the trunk cluster, and the rest as the branch cluster. The results showed that patients with higher heterogeneity had significantly poorer survival data compared to those with lower heterogeneity, with a median PFS of 30.0 weeks, compared to 60.0 weeks for patients with low tumor heterogeneity (HR, 2.9; P = 0.02). In addition, the TP53/PIK3CA/MTOR mutation appeared to have a significantly shorter median PFS in patients with a trunk cluster mutation (7.8 weeks, 95% CI, 7.4-26.8 weeks) than in patients with a branch cluster mutation (27.4 weeks, 95% CI, 11.8-63.5 weeks) and in patients without any mutation (HR, 4.5, 95% CI 1.2 to 17.6; P = 0.03) (17). These studies highlight the important role of ctDNA in breast cancer follow-up.

## 8 Conclusions and perspectives

**Figure 3:**
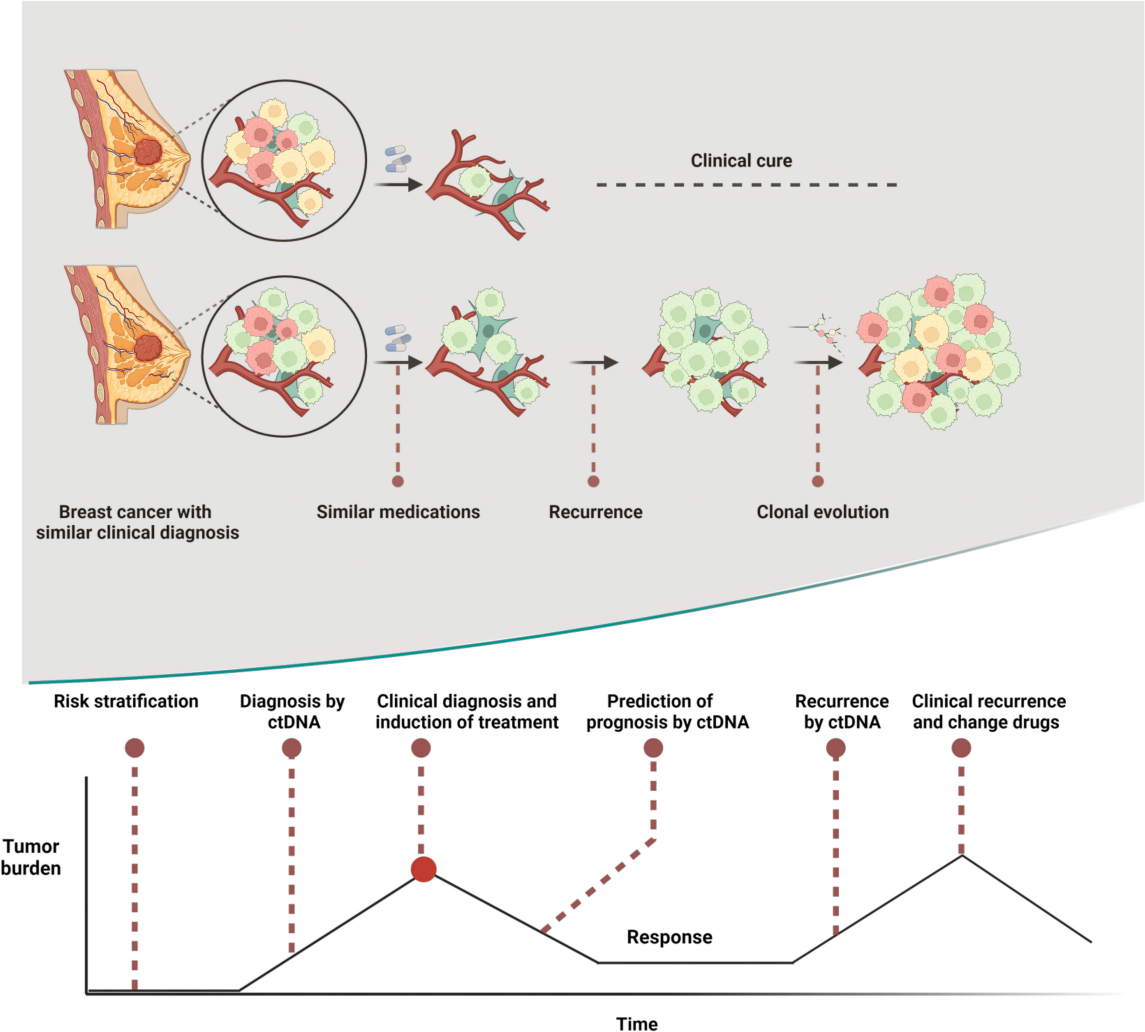
Heterogeneity is a major cause of prognostic differences in breast cancers with similar clinical profiles and ctDNA plays an important role at multiple stages in the breast cancer development curve. Created with BioRender.com.

Despite rapid advances in detection technology, we have not yet obtained a comprehensive understanding of breast cancer. ctDNA offers a direct window into breast cancer’s onset, progression, and metastasis, allowing us to overcome temporal and spatial heterogeneity and observe the complete evolution of a cancer clone. As a result, ctDNA has the potential to make significant contributions in various aspects of breast cancer management, including screening, diagnosis, prognosis, treatment, and follow-up (**Figure 3**). In the area of screening, we need to recognize its improved accuracy, but we also need to be concerned about whether it is too refined and leads to overtreatment. For patient follow-up, it is a key to identify the most appropriate targets and combinations among a large number of predictors in order to reflect as much information as possible while still balancing accuracy. In the area of diagnosis and prognosis, ctDNA has excelled, and although it is still rash to utilize it directly to determine whether breast cancer is diseased or not, new star genes continue to emerge that bring new directions to breast cancer diagnosis and treatment. For example, PIK3CA, preliminary results from the SOLAR-1 trial (ClinicalTrials.gov identifier: NCT02437318) presented at the 2018 San Antonio Breast Cancer Symposium suggest that ctDNA-based assessment of PIK3CA mutation status is a better indicator of PFS than tissue biopsy analysis (122). Subsequently, the FDA approved a companion diagnostic test based on the detection of PIK3CA mutations in the plasma of patients with advanced ER+/HER2- breast cancer. This is an important milestone in the movement of ctDNA towards large-scale clinical use.

Above, we have shown the role of ctDNA in breast cancer immunotherapy. So can the results of ctDNA testing guide breast cancer immunotherapy? Artemis (ClinicalTrials.gov identifier: NCT04803539) is a prospective phase II trial that is recruiting to see if ctDNA can be used to indicate intensification of therapy after adjuvant chemotherapy for non-metastatic TNBC. In this study, the results of ctDNA testing will be used to divide patients into an experimental group with carilizumab in combination with apatinib and capecitabine, and a control group with capecitabine only. Another similar study, Apollo (ClinicalTrials.gov identifier: NCT04501523), is looking to use the results of ctDNA testing, to see how it provides an answer to the question of whether patients with non-metastatic TNBC who have received NAC should receive capecitabine with or without tirilizumab as intensive therapy. It is hoped that the findings of these clinical trials will be as promising as their names suggest.

In addition, during the course of the review, we found that trying to evaluate many of the existing studies together was actually difficult because of the difficulty in harmonizing conditions for specimen processing and amplification prior to analysis across studies. A first large-scale external quality assessment of the impact of cfDNA quality, quantity and integrity showed that different extraction kits produced a wide range of cfDNA yields, which could vary by up to 100-fold. In fact, European organizations (CEN, SPIDIA4P) and international networks (CANCER-ID/European Union, BloodPAC/USA) are working on the development of standardized protocols for liquid biopsy methods in order to provide recommendations on technical specifications for the recommended handling, documentation and processing of blood specimens for ctDNA analysis (123). In addition, the characteristics of ctDNA, including the high degree of fragmentation and short half-life of the DNA mass itself, the detection limitations of low variant allele frequency in the background noise range, and the expensive cost of the assay are all issues that need to be resolved before ctDNA can be used in large-scale clinical applications.

Evidence for ctDNA as a valuable stand-alone assay rather than an optional one is growing, however, more evidence strong enough to support its feasibility for large-scale clinical use is still needed. Especially in early-stage breast cancer, although larger free DNA fragments are more frequently found in EBCs than in MBCs (124), the lack of quantity limits their ability to be detected as valuable. Perhaps with advances in detection technology, or perhaps with the advent of more rigorous large-scale clinical trials, the use of ctDNA testing in breast cancer can be taken to new heights, and we look forward to that day.

## Author contributions

JX: Writing – original draft. HG: Writing – review & editing. XG: Writing – review & editing. JM: Writing – review & editing. SD: Writing – review & editing. QL: Writing – review & editing. WY: Writing – review & editing.
